# Fine Needle Aspiration of Thyroid Nodules Using the Bethesda System for Reporting Thyroid Cytopathology: An Institutional Experience in a Rural Setting

**DOI:** 10.1155/2017/9601735

**Published:** 2017-02-09

**Authors:** Aili Guo, Yuuki Kaminoh, Terra Forward, Frank L. Schwartz, Scott Jenkinson

**Affiliations:** ^1^Ohio University-Heritage College of Osteopathic Medicine, Athens, OH 45701, USA; ^2^Department of Specialty Medicine, Athens, OH 45701, USA; ^3^The Diabetes Institute at Ohio University, Athens, OH 45701, USA

## Abstract

*Background.* Fine needle aspiration (FNA) remains the first-line diagnostic in management of thyroid nodules and reduces unnecessary surgeries. However, it is still challenging since cytological results are not always straightforward. This study aimed to examine the results of thyroid FNA using the Bethesda system for reporting thyroid cytopathology (TBSRTC) to establish the level of accuracy of FNA procedures in a rural practice setting. *Method.* A retrospective chart review was conducted on existing thyroid FNA performed in a referral endocrine center between December 2011 and November 2015. *Results.* A total of 159 patients (18–88 years old) and 236 nodule aspirations were performed and submitted for evaluation. 79% were benign, 3% atypia/follicular lesion of unknown significance (AUS/FLUS), 5% follicular neoplasm/suspicious for follicular neoplasm (FN/SFN), 4% suspicious for malignancy (one case was indeed an atypical parathyroid neoplasm by surgical pathology), 2% malignant, and 7% nondiagnostic. Two cases also had advanced molecular analysis on FNA specimens before thyroidectomy. *Conclusion*. The diagnostic yield of FNA cytology from our practice in a rural setting suggests that accuracy and specificity are comparable to results from larger centers.

## 1. Introduction

Palpable thyroid nodules are a common finding, occurring in 3–7% of the population, and the estimated annual incidence rate of 0.1% in the United States suggesting of 300,000 new nodules is detected in this country every year [1–3]. The majority of these nodules are benign, but malignancy is found in approximately 5–15% of cases depending on age, sex, radiation exposure history, family history, and other factors warranting further evaluation [4–6]. Since most patients with thyroid nodules are asymptomatic, clinical and thyroid ultrasound risk factors for malignant disease are routinely reviewed to determine necessity for further thyroid fine needle aspiration (FNA) [7]. Ultrasound-guided thyroid FNA followed by cytological examination is considered the standard care due to its cost effective and minimally invasive nature. Interpretation of FNA results thus becomes the key step in order for clinicians to advise if more invasive evaluation is necessary.

The Bethesda system for reporting thyroid cytopathology (TBSRTC) resulted from a conference held at the National Institutes of Health in 2007 [8, 9]. Up to now, clinical management of the “indeterminate” cell types, that is, cytological categories of atypia/follicular lesion of undetermined significance and follicular neoplasm/suspicion for a follicular neoplasm, still poses the biggest challenge of waiting with repeat biopsies versus diagnostic surgery for a definitive diagnosis. Use of molecular marker testing on FNA samples may be complement to FNA procedures when cytological results are “indeterminate”; however, the approach can be costly and the sensitivity of available tests can be improved. Whilst the majority of the previous published literature in thyroid FNA studies is from large urban and suburban academic institutions employing multiple practitioners, there is a paucity of data from rural areas.

TBSRTC has been used in our institution during the study interval. The purpose of the present study is to examine the outcome of FNA of thyroid nodules by using TBSRTC from our single academic endocrine institution, mostly serving Appalachian southeastern Ohio, and to establish the level of accuracy of FNA in this rural setting. Specifically, the study is to examine the incidence rates of thyroid cytological categories and the inadequate sampling rate from our practice in comparison to that from the literature. The study further analyzed the concordance rate between FNA cytology and surgical pathology.

## 2. Method

### 2.1. Study Design and Thyroid FNA Procedures

A retrospective chart review within our electronic medical record (EMR) system was used to collect data on patients evaluated for thyroid nodules between December 2011 and November 2015 by an endocrinologist in a referral endocrine/diabetes center in Athens, Ohio, serving as a referral center for a population of approximately 200,000 people. The majority of patients undergoing FNA were referred by primary care providers, whilst a minority was referred for thyroid FNA after evaluation by ENT surgeons who do not routinely perform thyroid surgeries.

Data collected included reports of thyroid FNA cytology; for patients undergoing thyroid surgeries, also included are surgical pathology, blood thyroid function tests, sonographic characteristics of thyroid nodules, and molecular marker testing of FNA specimen if performed. Data were deidentified before the analysis. Unless otherwise specified, multiple visits and repeat FNA cytology on the same patient were treated as independent incidences.

Ultrasound-guided thyroid FNA procedures were performed using 27 G or 25 G needles with 3–5 passes per each nodule under real-time ultrasound guidance as an outpatient procedure with topical anesthesia cream for patient comfort. Patient coagulation profiles were not routinely obtained prior to biopsy. However, patients on anticoagulation therapy were asked to stop taking their medication for 5 to 7 days prior to biopsy if possible.

Aspirated specimens were handed over to on-site board certified pathologist in the ultrasound biopsy room to evaluate the adequacy of aspirated specimens. Slides were stained with Wright stain and Papanicolaou stain. The Ohio University Institutional Review Board approved this study for IRB exemption.

### 2.2. Thyroid FNA Cytology Categories

Initial cytological results from the thyroid FNA procedures were classified into six diagnostic categories based on the TBSRTC system: (I) nondiagnostic, (II) benign, (III) atypia/follicular lesion of undetermined significance (AUS/FLUS), (IV) follicular neoplasm/suspicion for a follicular neoplasm (FN/SFN), (V) suspicious for malignant papillary thyroid carcinoma (SPTC), and (VI) malignant or PTC. A second expert opinion was sought in most cases with initial FNA cytology falling into “indeterminate” (AUS/FLUS and FN/SFN) categories and in a few cases with other categories, desired by the pathologist, at a large academic medical center. There were two cases that had further molecular marker testing on thyroid FNA specimens. For cases where the second opinion was discordant with the initial cytological report, the classification that yielded the worse prognosis was used for the purposes of this study. In cases whose nodules were not surgically removed, further repeated FNA cytological results or stabilities of thyroid nodules on follow-up ultrasonography up to November 2015 were the reference criteria for the final diagnosis.

### 2.3. Surgical Pathology Results

In cases that underwent thyroidectomy or thyroid lobectomy, the final diagnoses were determined by the reports of surgical pathology. These cases were regrouped after thyroid surgeries into the following categories: (1) benign, including follicular adenoma, hyperplastic nodule, colloid/nodular goiter, and thyroiditis, and (2) malignant lesion, including PTC, follicular carcinoma, and atypical parathyroid neoplasm. The concordance rates between FNA cytology and surgical pathology were further analyzed. In cases where a second expert opinion was sought, concordance rates to the surgical pathology were compared between the initial and the second opinions of cytological diagnoses.

### 2.4. Statistical Analysis

Data was presented as mean ± SD. Chi-square test or Fisher's exact test was used to compare the features of benign and malignant masses via two-way ANOVAs along with *t*-tests for select comparisons. Results were considered significant if *p* < 0.05.

## 3. Results

### 3.1. Cytology Results of Thyroid FNA Biopsies

A total of 159 patients, including 138 females (87%, mean age 52.4 years) and 21 males (13%, mean age 57.2 years), were seen for thyroid nodule evaluations during the study period. These patients had 236 nodule FNAs performed over 177 visits that were submitted for evaluation. As shown in Table 1, results from cytological reports were classified using TBSRTC: (I) nondiagnostic 16/236 (7%), (II) benign 186/236 (79%), (III) AUS/FLUS 8/236 (3%), (IV) FN/SFN 12/236 (5%), (V) SPTC 10/236 (4%), and (VI) malignant or PTC 4/236 (2%).

### 3.2. Comparison of Surgical Pathology with Cytology Results

There were total of 25/159 cases (16%) who underwent thyroid surgery after FNA procedures, including 5 cases with benign and 1 case with nondiagnostic cytological results for symptomatic nodular goiter, 3 cases with AUS/FLUS, 7 cases with FN/SFN, and 9 cases with PTC or SPTC. Of note, one elderly with PTC cytology who declined surgery secondary to comorbidities was not included in this further analysis.

As shown in Table 2, all 6 cases with either benign or nondiagnostic FNA cytology were confirmed to have benign disease by surgical pathology. Among the “indeterminate” cytological categories, out of 3 cases of AUS/FLUS, 2 were benign and 1 was PTC by surgical pathology; and out of 7 cases of FN/SFN, 3 were malignant PTC, 1 case was minimal invasive follicular carcinoma, and 3 cases were benign by surgical pathology. Out of 3 cases of malignant PTC, 2 cases were malignant PTC by surgical pathology, whereas 1 was benign. Thus, the sensitivity and specificity of FNA diagnostic accuracy were 100% and 67% for category VI malignant and 100% and 83% for category V SPTC, respectively. Among the 6 cases of SPTC, 4 were confirmed to be malignant PTC after surgery, 1 case was benign, and 1 case was diagnosed as atypical parathyroid neoplasm by surgical pathology. This left 2.4 × 2.2 × 2.0 cm complex nodule was identified in the midportion of left thyroid lobe by thyroid ultrasound study. During operation, the left thyroid nodule was found to be densely adherent and encasing the left recurrent nerve. Surgical pathology was reported as “(left) atypical parathyroid tumor with malignancy potential” based on findings that hyperplastic parathyroid infiltrates the adjacent thyroid follicles with focal areas of calcifications and surrounding oncocytic cells at some area; however, mitotic figures in the proliferating parathyroid cells are rare or not seen.

Among the 25 cases with surgical pathology, 12 cases were malignant and 13 cases were benign, respectively. The preoperative TSH levels were not significantly different between the surgical malignant (mean ± SD, 4.03 ± 4.80) and nonmalignant (2.09 ± 2.14, *p* value = 0.155) groups.

### 3.3. Comparison of Concordance Rates to Surgical Pathology between Initial Thyroid FNA Cytology and Second Expert Opinion of Diagnoses

There were 33 FNA specimens referred for a second expert opinion, out of which 20 nodules were surgically removed. As shown in Table 3, when compared to surgical pathology, the initial cytology was in agreement in 15/20 (75%) specimens, which is comparable to that of second expert opinions. The concordant rate between initial cytology and second expert opinions was 55% (11/20 nodules).

### 3.4. Outcomes of Two Cases with Further Molecular Marker Testing

There were two cases with “intermediate” cytological results where further molecular markers were tested on thyroid FNA specimens using the Afirma Gene Expression Classifier (Afirma GEC, Veracyte, South San Francisco, CA 94080). The first case was a 54-year-old otherwise healthy Caucasian woman with a 2.9 cm heterogeneous nodule, mostly isoechoic without calcifications in the right thyroid lobe on thyroid ultrasound study. She was asymptomatic and euthyroid but with positive TPO-ab, evident for Hashimoto's thyroiditis. The initial FNA cytology was read as “consistent with PTC,” but a second expert opinion rereviewed as “atypical (AUS).” She was then referred for surgical consultation at a large academic center, where a thyroid FNA procedure was repeated and read as “SFN.” Molecular markers were simultaneously tested on FNA specimen by Afirma GEC assay and reported as “suspicious.” She subsequently underwent a total thyroidectomy, and the surgical pathology concluded as a benign follicular adenoma (oncocytic type) with background chronic lymphocytic thyroiditis.

The second case was a 62-year-old euthyroid Caucasian female with medical history significant for coronary artery disease status postmyocardial infarction. She was referred to our center by primary care physician for a second opinion on management of a large thyroid mass. Apparently, a multinodular goiter was incidentally identified on her chest imaging study and the initial thyroid FNA cytology of a right 3.9 cm heterogeneous nodule was read as “SFN.” In order to decide if an active surveillance management approach can be an alternative to immediate surgery with her cardiac condition, a second thyroid FNA procedure was performed in our clinic and specimens were sent out for additional molecular marker testing by Afirma GEC assay. The gene expression analysis fell into the same category of the first case, “suspicious”; but her final surgical pathology of total thyroidectomy revealed a minimal invasive follicular carcinoma.

## 4. Discussion

Thyroid cancer is the most common endocrine cancer with growing incidence worldwide. More recent studies indicated that the yearly incidence has nearly tripled from 4.9 per 100,000 in 1975 to 14.3 per 100,000 in 2009, corresponding to approximately 63,000 new cases of thyroid cancer which were predicted to be diagnosed in 2014 [7] compared with 37,200 in 2009 when the last ATA guidelines were published [4]. At present, thyroid FNA cytology is still the most accurate and cost-effective method for evaluating thyroid nodules. A uniform reporting system for thyroid FNA will facilitate effective communication among health care providers, facilitate cryptologic-histologic correlation for thyroid diseases, and allow easy and reliable sharing of data from different laboratories for national and international collaborative studies. TBSRTC has been widely used since its publication [8, 9]. The diagnostic yield of FNA cytological results according to TBSRTC from our single academic institute in a rural setting was 93% overall, with 7% nondiagnostic, 79% benign, 8% follicular lesion (AUS/FLUS or FN/SFN), 4% suspicious for malignancy, and 2% malignant, respectively. These results are comparable to the previously published studies [10–13]. Of note, with on-site assessment of adequacy at the time of sampling by an experienced pathologist, we have a relatively lower rate in nondiagnostic category in comparison to individual studies ranging from 7 to 20% from a large multicenter study, and the yield was comparable to centers with higher procedure volume (Table 1). Previous studies have reported nondiagnostic FNAs as having malignancy rates ranging 2–11.4% [14–16]. Since our current dataset is small and limited by the narrow scope of follow-up for repeat FNA, this study would not be able to address this question.

There were a total of 25 out of 159 cases (16%) with histological follow-up after thyroid FNA procedures. In majority of cases, the FNA diagnosis was in concordance with final surgical pathology. Among the 4 cases with PTC cytology nodules, whilst 1 elderly case chose no surgery secondary to her comorbidities, 2 (67%) were confirmed to be papillary thyroid cancer and the other case was benign by surgical pathology. In the group of SPTC cytology, 4 out of 6 cases (67%) were indeed confirmed to be malignant papillary thyroid cancer and 1 was benign. It is noteworthy that, concerning the remaining 62-year-old female case with SPTC cytology, the surgical pathology turned out to be an atypical parathyroid neoplasm. Preoperative detection of a parathyroid adenoma can sometimes be challenging. There is significant overlap in the cytomorphologic features of cells derived from parathyroid and thyroid gland, although previous study suggested that the presence of stippled nuclear chromatin, prominent vascular proliferation with attached epithelial cells, and frequent occurrence of single cells and naked nuclei are useful clues that favor parathyroid origin [17]. Parathyroid hormone (PTH) assays on the needle washout of FNA specimen of suspected parathyroid tissue are further tools of localizing parathyroid adenomas [18, 19]. Immunostaining for PTH performed on Pap smears or cell block sections may be valuable for confirming thyroid origin of the cells prior to surgery.

Follicular thyroid carcinomas and papillary thyroid carcinomas are classified as differentiated thyroid carcinomas (DTC), comprising approximately 90% of all thyroid cancers, of which approximately 14–25% are follicular thyroid carcinoma [7]. Cytological differentiation of follicular thyroid carcinoma from PTC includes confirmation of follicular cells lacking nuclear atypia seen in PTC. Meanwhile, continued efforts have been made to further separate benign versus malignant lesions in the category of “indeterminate” results, including molecular marker testing on FNA specimens. The “rule-in” principle test determines the presence of single gene point mutations (BRAF or RAS) or gene rearrangements (RET/PTC, PAX8/PPAR*γ*), whilst the other one uses a 167-gene expression classifier (Afirma GEC) to assess for benign characteristics as a “rule-out” test [20, 21]. In our cohort, two cases with “indeterminate” cytology had molecular marker testing prior to thyroidectomy. The results of both cases by Afirma GEC were “suspicious”; however, the surgical pathological findings concluded one was benign follicular adenoma (oncocytic type) and the other was malignant minimal invasive follicular carcinoma. Our data is in agreement with the statement of the 2015 American Thyroid Association (ATA) [7, 22] and the 2010 American Association of Clinical Endocrinologists (AACE) [3] management guidelines; that is, long-term outcome data proving routine clinical utility are needed.

Seeking a second opinion for cytology is a common practice when thyroid nodule specimens are classified as “indeterminate” and is considered to be an appropriate additional step in helping to achieve an accurate diagnosis [23]. The concordant rate between initial cytology and second expert opinions was 55% (11/20 nodules), reflecting an inherent limitation to the reproducibility of interpreting any cytology specimen as demonstrated by a blinded prospective evaluation of interobserver concordance using Bethesda classification [24]. When compared with final surgical pathology results, the initial cytology concordance rate was 75% (15/20 nodules), which is not statistically different from that of second opinions (70%, 14/20 nodules) of FNA diagnoses, demonstrating effectiveness and adequacy of our FNA practice.

We recognized that our study has a number of limitations due to the nature of a retrospective study and the relatively small numbers. Nevertheless, our data from a single center has provided insight into the care of rural populations, which is comparable to the results from most published studies involving large patient populations in major academic centers. Coupling with clinical and biochemical evaluation, ultrasound-guided FNA remains the first-line diagnostic procedure in the management of thyroid nodules in our practice. Our study shows that FNA cytology of thyroid nodules can be performed and interpreted reliably by qualified physicians in rural clinics similar to that seen in major urban teaching hospitals which offer significant savings of both time and cost to patients.

## Figures and Tables

**Table 1:** Cohort FNA classification by the Bethesda system for reporting thyroid cytopathology (TBSRTC).

TBSRTC category	Cohort incidence *n* = 236 (%)	TBSRTC expected incidence [8]	Published incidence by using TBSRTC [10–13]
I—nondiagnostic	16 (7%)	<10%	2–24%
II—benign	186 (79%)	60–70%	39–77%
III—atypia/follicular lesion of unknown significance (AUS/FLUS)	8 (3%)	<7%	0.8–27%
IV—follicular neoplasm or suspicious for follicular neoplasm (FN/SFN)	12 (5%)	N/A	1–25%
V—suspicious for malignancy (SPTC)	10 (4%)	N/A	1–6%
VI—malignant (PTC)	4 (2%)	3–7%	2–16%

**Table 2:** Comparison of thyroid cytology and surgical pathology.

FNA cytology	Resected cases (*n* = 25)	Surgical pathology	
		Benign (*n* = 13)	Malignant (*n* = 12)
Nondiagnostic	1	1 (100%)	0
Benign	5	5 (100%)	0
Atypia/follicular lesion of unknown significance (AUS/FLUS)	3	2 (67%)	1 (33%) PTC
Follicular neoplasm or suspicious for follicular neoplasm (FN/SFN)	7	3 (43%)	4 (57%) 3—PTC, 1—follicular carcinoma
Suspicious for malignancy (SPTC)	6	1 (17%)	5 (83%) 4—PTC, 1—parathyroid neoplasm with malignant potential
Malignant (PTC)	3^∗^	1 (33%)	2 (67%) PTC

^∗^One case declined surgery due to age and comorbidities and disease is stable at the time of follow-up.

**Table 3:** Comparison of concordant results to surgical pathology between the initial and expert opinions of cytological reports.

Thyroid nodules (*n* = 20)	Concordant to surgical pathology	Disconcordant to surgical pathology
The initial cytology by local pathologist	15 (75%)	5 (25%)
The expert opinion by outside pathologist(s)	14 (70%)	6 (30%)

## Abbreviations

AUS: Atypia of undetermined significance
DC: Diagnostic cytology
DTC: Differentiated thyroid carcinoma
FLUS: Follicular lesion of unknown significance
FN: Follicular neoplasm
FNA: Fine needle aspiration
FTC: Follicular thyroid carcinoma
PTC: Papillary thyroid carcinoma
SPTC: Suspicious for malignant papillary thyroid carcinoma
TBSRTC: The Bethesda system for reporting thyroid cytopathology
TPO-ab: Thyroid peroxidase antibodies.
